# Crosstalk between microRNA and Oxidative Stress in Heart Failure: A Systematic Review

**DOI:** 10.3390/ijms232315013

**Published:** 2022-11-30

**Authors:** Dominika Klimczak-Tomaniak, Julia Haponiuk-Skwarlińska, Marek Kuch, Leszek Pączek

**Affiliations:** 1 Department of Cardiology, Hypertension and Internal Medicine, Medical University of Warsaw, 02-091 Warsaw, Poland; 2 Department of Pediatric Cardiology and General Pediatrics, Doctoral School, Medical University of Warsaw, 02-091 Warsaw, Poland; 3 Department of Immunology, Transplantation and Internal Medicine, Medical University of Warsaw, 02-091 Warsaw, Poland

**Keywords:** oxidative stress, microRNA, heart failure, myocardial remodeling, myocardial hypertrophy, mitochondria, ROS

## Abstract

Heart failure is defined as a clinical syndrome consisting of key symptoms and is due to a structural and/or functional alteration of the heart that results in increased intracardiac pressures and/or inadequate cardiac output at rest and/or during exercise. One of the key mechanisms determining myocardial dysfunction in heart failure is oxidative stress. MicroRNAs (miRNAs, miRs) are short, endogenous, conserved, single-stranded non-coding RNAs of around 21–25 nucleotides in length that act as regulators of multiple processes. A systematic review following the PRISMA guidelines was performed on the evidence on the interplay between microRNA and oxidative stress in heart failure. A search of Pubmed, Embase, Scopus, and Scopus direct databases using the following search terms: ‘heart failure’ AND ‘oxidative stress’ AND ‘microRNA’ or ‘heart failure’ AND ‘oxidative stress’ AND ‘miRNA’ was conducted and resulted in 464 articles. Out of them, 15 full text articles were eligible for inclusion in the qualitative analysis. Multiple microRNAs are involved in the processes associated with oxidative stress leading to heart failure development including mitochondrial integrity and function, antioxidant defense, iron overload, ferroptosis, and survival pathways.

## 1. Introduction

### 1.1. microRNA

MicroRNAs (miRNAs, miRs) are short, endogenous, conserved, single-stranded non-coding RNAs of around 21–25 nucleotides in length [1]. miRNAs, when transcribed as precursors and processed into mature miRNA, can interact with target cellular messenger RNA (mRNA) transcripts [2]. In most cases, they induce target degradation. Through gene expression regulation, miRNAs have an important role in various cellular processes such as cell growth, proliferation, differentiation, apoptosis, and stress response [3,4,5]. The latest miRNBase (v22.1) contains microRNA sequences from 271 species: 38,589 precursors and 48,860 mature microRNAs. To date, the base includes 1917 precursors and 2654 mature sequences described in humans [6].

### 1.2. Oxidative Stress

Oxidative stress is recently widely described as an important factor in the pathophysiology of many human diseases [7,8,9]. It is caused by the overproduction of reactive oxygen species (ROS) which are the byproducts of normal cellular metabolism. ROS include both radicals and non-radicals, such as superoxide anions, hydrogen peroxide, hydroxyl ion, alkoxyl, singlet oxygen, ozone, and peroxyl radicals. Furthermore, because of their unique characteristics, excess ROS can not only trigger many molecular implications including inflammation and programmed cell death, but also target, terminally modify, and damage different molecules in the cell including proteins, lipids, and nucleic acids [10,11]. Oxidative stress has been widely described as a pathogenesis agent, diagnostic tool, and therapeutic target in medical conditions, including cardiovascular diseases [12].

#### 1.2.1. ROS Origin, Formation, and Function

ROS are most often the result of the phenomenon described as free radical leak which is observed in mitochondria. In fact, mitochondria play a fundamental role in the cellular physiology by synthesizing adenosine triphosphate (ATP) in the process of Electron Transport Chain (ETC). The ETC contains four complexes (Complexes I–IV) embedded in the inner mitochondrial membrane (IMM), and two small electron carriers, ubiquinone (Q) and cytochrome (C), which carry electrons between the complexes until they are passed to the final electron acceptor—the oxygen. However, not all electrons within the ETC are transferred to the oxygen due to the free-radical leak. This results in the production of endogenous, mitochondrial ROS, mostly superoxide radical anion and H_2_O_2_ [11,12,13]. The accumulation of ROS inside mitochondria can provoke the process called mitochondrial permeability transition pore (mPTP) opening which results in the release of the ROS to the cytoplasm of the cell [14] and its activity there. Nonetheless, there are more mechanisms of mitochondrial ROS production described [15].

Non-mitochondrial ROS are produced with a key contribution of several enzymes including myeloperoxidase (MPO), xanthine oxidase (Xo), uncoupled nitric oxide synthase (NOS), lipoxygenase, and nicotinamide adenine dinucleotide phosphate (NADPH) oxidase (NOX), which is considered as a significant source of ROS. The ROS-containing nitrogen are often distinguished as reactive nitrogen species (RNS). RNS in general are important in pathogenesis of cardiovascular events [12,16]. Finally, exogenous ROS can also be formed after the environmental factors exposure such as pollutants, cigarette smoke, ultraviolet (UV) radiation, and xenobiotics [17].

Despite the toxic effect and participation in pathogenesis of many diseases, it is worth mentioning that ROS generation is a natural part of aerobic life. Basal levels of ROS are essential for signaling function, defense against microorganisms, gene expression, growth, and death. Living organisms are equipped with mechanisms of ROS release control, including enzymatic e.g., superoxide dismutase (SOD), catalase (CAT), and non-enzymatic means e.g., ascorbate, beta-carotene.

#### 1.2.2. microRNA and Oxidative Stress in Cardiovascular Diseases

Today, sophisticated methods of redox signaling pathways investigation allow the inquiry into the pathogenesis of many diseases, including widely discussed cardiovascular events and HF, to which ROS species may contribute [12]. There is evidence of interactions between cardiac miRNAs and ROS in different cardiovascular events including, e.g., atherosclerosis, myocardial infarction, diabetic cardiomyopathy, and chemotherapy-induced cardiomyopathy mainly in animal models [18]. In response to increased ROS concentration, the cardiovascular diseases can be initiated and developed by fibrosis, apoptosis, necrosis and proliferation or hypertrophy of both smooth and cardiac muscles, endothelial cells, and cardiac fibroblasts. It was proven that microRNAs are involved in these processes [19,20,21]. Moreover, some microRNAs have been identified as regulators of oxidative stress in cardiovascular system by interaction with ROS generators, antioxidant effectors, and signaling pathways [22].

This review presents current knowledge concerning the miRNA role involved in oxidative stress in the development of the heart failure. To our knowledge, no systematic reviews concerning miRNA and oxidative stress in heart failure have been published to date.

## 2. Materials and Methods

A computer search was conducted according to the Preferred Reporting Items for Systematic Reviews and Meta-Analyses (PRISMA) scheme by two independent observers in four major databases (Pubmed, Embase, Scopus, Scopus direct) using the following search terms: ‘heart failure’ AND ‘oxidative stress’ AND ‘microRNA’ or ‘heart failure’ AND ‘oxidative stress’ AND ‘miRNA’. The PRISMA statement consists of a 27-item checklist and a four-phase flow diagram whose aim is to help authors in the reporting of systematic reviews and meta-analyses [23].

**Figure 1:**
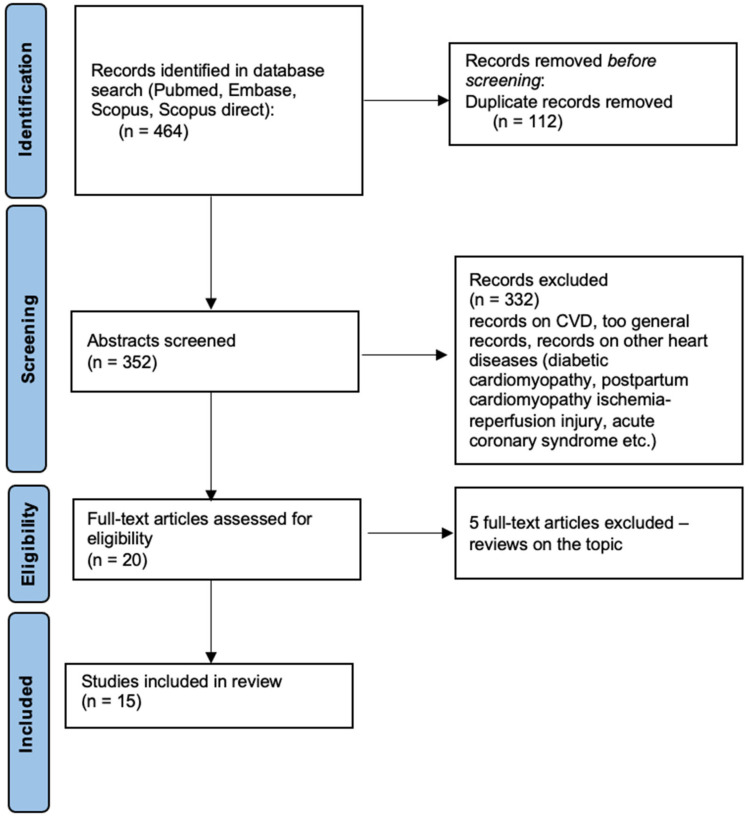
Flowchart summarizing the literature search methodology.

After the database research, the obtained abstracts were screened by the authors and only the articles connected to the topic of microRNA, oxidative stress, and heart failure were included. Articles about cardiovascular diseases (CVD) in general, postpartum cardiomyopathy, diabetic cardiomyopathy, ischemia-reperfusion damage, and acute coronary syndromes were excluded. Finally, only 15 articles were outlined after the removal of 5 reviews on the topic. The methodology of the database research is summarized in Figure 1.

## 3. Results

The final number of full-text original articles included in the analysis was *n* = 15 (Figure 1). The full list of relevant articles on this topic is listed in the Table 1. Below we explore the current evidence in detail.

Heart failure is defined as a clinical syndrome consisting of key symptoms and is due to a structural and/or functional alteration of the heart that results in increased intracardiac pressures and/or inadequate cardiac output at rest and/or during exercise [38]. One of the key mechanisms that determine the dysfunction of the heart muscle in heart failure is oxidative stress and mitochondrial dysfunction [39,40]. To date, several miRNAs have been identified and characterized in the heart failure basing on the animal models including: miRNA-27a, miRNA-28-3p, miRNA-34a, miR-122, miRNA-195-5p, miRNA-224-5p, miRNA-296-3p, miRNA-345-3p, miRNA-532, miRNA-671-5p, miRNA-690, miRNA696, miRNA-1306-5p, and miRNA-3082-5p (Table 1).

### 3.1. Mitochondrial Integrity and Function

**Figure 2:**
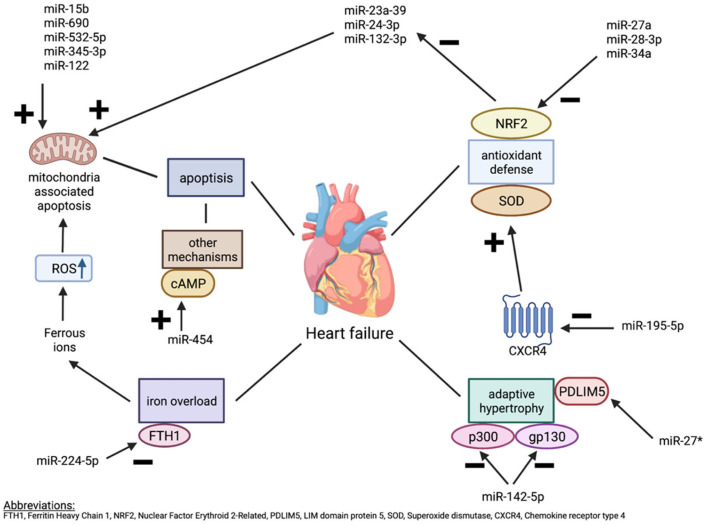
The balance between the apoptosis induced by mitochondrial oxidative stress and antioxidant defense mechanisms. (*—passenger strand).

As described earlier, ROS formation strongly depends on the mitochondrial function. Wang, X. et al. (2017) [24] and Shi, Y. et al. (2021) [25] described association between microRNA and mitochondrial dysfunction contributing to cardiomyocyte apoptosis, and consequently heart failure. Wang et al. evaluated mitochondrial expression of microRNA in samples of mitochondrial RNA coming from animal model of HF (transverse aortic constriction (TAC) at early (4 weeks) and late (8 weeks) phase). The authors report on four microRNAs enriched in mitochondria of the failing heart (mmu-miR-690, mmu-miR-532-5p, mmu-miR-696, and mmu-miR-345-3p) (Figure 2). The pathway analysis revealed the association of this microRNAs with mitochondria biogenesis and fission, fatty acids metabolism, oxidative stress, and apoptosis [24]. Next, Hand2 transcription factor normally plays an essential role in cardiac morphogenesis and its disruption can cause impaired cardiac development, apoptosis of cardiomyocytes, and cardiac hypertrophy. miR-122 was found to inhibit Hand2 and consequently increase dynamin-related protein-1-mediated excessive mitochondrial fission which results in cardiomyocyte apoptosis.

The transgenic mice with excessive miR-122 expression not only showed macroscopically enlarged hearts with lowered contractile function but most importantly the Western blot quantification, immunofluorescence, and histochemical staining showed increased level of Dpr1 protein and mRNA in comparison to healthy controls. These results suggested that miR-122 overexpression leaded to cardiomyocyte apoptosis by mitochondrial fission, contributing to heart failure development in these transgenic mice [25] (Figure 2).

miRNA-15b is another microRNA species connected to the mitochondrial integrity and function in cardiomyocytes. In fact, suppression of dicer endonuclease, critical for maturation of miRNA results in rapid loss of cardiac function in mice model. MiRNA-15b was found to silence Pim-1 kinase, required for mitochondrial functioning and integrity of cardiac muscle (Figure 2). Authors analyzed mitochondrial structure by transmission electron microscope (TEM) and observed a loss of mitochondrial structural integrity in cardiac tissue represented by matrix swelling and unfolded cristae from Dicer^−/−^ mice. Therefore, they concluded that targeting miRNA-15b could serve as a future therapeutic approach in the patients with HF [26].

### 3.2. Antioxidant Defence

Nuclear factor erythroid 2-related factor 2 (Nrf2), an important antioxidant defense mechanism closely associated with oxidative stress-mediated cardiac remodeling in chronic heart failure (CHF), was also found to be inhibited by increased microRNA-27a and microRNA-28-3p. The microRNA-34a expression in the infarcted hearts contributed to decrease of Nrf2-targeted antioxidant enzymes (Figure 2). These microRNAs were preferentially incorporated into exosomes and secreted into the extracellular space in which microRNA-enriched exosomes mediated intercellular communication and Nrf2 dysregulation, which may play role in the setting of CHF [27]. On the other hand, the protective role of microRNA-27a has been reported as well. A study by Tian et al. evidenced that Kruppel-like factor 5 (KLF5), a pro-apoptotic member of the Kruppel-like transcription factor family, induces cell and tissue damage in myocardial infarction (MI) through downregulation of miR-27a and the subsequent activation of GFPT2/TGF-β/Smad2/3 axis (Figure 2) [31]. Some recently published data describe the role of miRNA-27a passenger strand (miRNA-27a*) especially as the hypertrophy mediator in post MI-hearts in a mechanism described below (see Section 3.4) [27,41]. Further on, Hua et al. described microRNA-34a role in cardiovascular fibrosis, and Smad4/TGF-β1 signaling. It was reported that the inhibition of miR-34 reduced cellular reactive oxygen species (ROS) through the miR-34a/SIRT1 signaling pathway, but also through alleviation of the Kruppel-like factor 4 (KLF4). Next, miRNA-34a eased oxidative stress (induced by H_2_O_2_) in protection of vascular endothelial cells by pointing SIRT1 (Figure 2) [42]. Based on the data above, it is clear that microRNA exert multidirectional effects, besides their effect on oxidative stress.

Oxidative/nitrosative stress has also been described as a trigger of heart dysfunction and remodeling. Activation of nitric oxide-cyclic guanosine monophosphate-protein kinase G signaling by sildenafil improves cardiac remodeling in a mouse model of pressure-overload-induced (PO-induced) heart failure. Sildenafil is a selective inhibitor of type 5 phosphodiesterase that provides positive effects on hemodynamics in pulmonary hypertension (PH). This drug has been approved for use in primary idiopathic PH, but it also is often utilized empirically in heart failure (HF), because it frequently results in PH [43]. Lewis et al. [44] found that sildenafil upregulated Nrf2, an important antioxidant defense player, by inhibiting the maturation of PO-induced miRNAs, such as miR-23a-3p, miR-24-3p, and miR-132-3p (Figure 2). These miRNAs govern the transition from compensated hypertrophy to decompensated hypertrophy and contribute to mitochondrial metabolism and redox homeostasis dysregulation. R–like endoplasmic reticulum (ER) kinase (PERK) was identified to be crucial for sildenafil-mediated improvement of mitochondrial dysfunction in failing hearts through the suppression of HF-induced miRNAs. All in, the suppression of the above miRNAs play a vital role in sildenafil-induced cardiac protection [44]. However, the data come from animal study and the results from human studies do provide ambiguous information on the effects of sildenafil treatment. Whereas sildenafil has been shown to improve hemodynamics in patients with HFrEF [45], potential adverse effects of sildenafil on mitochondrial function and endoplasmic reticulum stress in HFpEF patients have been reported [44,45]. Therefore, further studies in HFpEF and HFrEF patients are necessary to clarify the difference in its effects.

### 3.3. Iron Overload and Ferroptosis

Iron is an element essential for various enzymatic reactions and therefore maintaining normal cardiac function. However, excess iron in the cardiomyocytes leads to the increase of ROS formation, thus generating oxidative damage to large molecules such as DNA, membrane lipids and proteins [46]. Ferroptosis is an iron-dependent form of cellular death in which ferric iron (Fe^3+^) from Fenton reaction increases ROS concentration. In ferroptosis, ROS activate lipoxygenases which damage cellular membranes. It is generally a morphologically different process from apoptosis or necrosis while it manifests as dense mitochondria with loss of cristae and previously mentioned cellular membranes damage. Glutathione peroxidase 4 (GPX4) was described several times as an endogenous mechanism of ferroptosis alleviation [47] and may be an important factor regarding possible therapeutic modalities. Ferroptosis has been observed in the cell death as pathogenesis in many human diseases, also in heart diseases including ischemia/reperfusion damage in vitro studies [47].

Zheng et al. [30] report that Ferritin Heavy Chain 1 (FTH1) gene was markedly downregulated in mice with TAC, a method to induce acute heart failure, which in turn results in a release of a large amount of ferrous ions into the cytoplasm. In the above study, circular RNAs (circRNAs), mainly circSnx12, were the targeted molecules, but its correlation with miR-224-5p in heart failure mechanism was found. The authors identified a protein associated with iron metabolism (FTH1), predicted that miR-224-5p might bind to FTH1 3′UTR by TargetScan and RegRNA 2.0 database, and further determined that miR-224-5p could be a potential binding target for circSnx12 through starBase database, which was proven in their results. It was observed that high miR-224-5p expression can downregulate FTH1 expression, and therefore contribute to iron overload in myocardial cells, leading to cardiac cell death in the mechanism of ferroptosis [30]. The literature on miRNA and the role of ferroptosis in human heart failure is very limited, and therefore merits further exploration.

### 3.4. Cardiac Hypertrophy and Remodelling

One of the key pathophysiological components of the development and progression of the heart failure is the increased systemic pressure which increases the workload that the left ventricle must withstand. Therefore, the myocardium experiences structural and functional changes to comply with the increased demand resulting in the cardiac hypertrophy and/or remodeling [48]. The miRNAs that up to today have been correlated with the myocardial hypertrophy and remodeling in animal models include: miRNA-21-3p, miRNA-27a, miRNA-93miR216-5p, miRNA-142-3p, miRNA-142-5p, miRNA219a-2-3p, miRNA381-3p, miRNA466c-5p, miRNA542-3p, and miRNA-702-5p (Table 1).

The repression of miR-142-5p was found to be a crucial element of adaptive hypertrophy in response to hemodynamic and oxidative stress. As a result of physiologic repression of miR-142-5p transduced by mitogen-activated protein kinases (MAPK) and acetyltransferase p300, myocardial growth (mediated by p300) and activation of myocyte survival pathways (mediated by cytokine receptor gp130) are possible as both p300 and gp130 are direct targets of miR-142-5p (Figure 2). The cumulative effect is activation of cytokine-mediated survival signal pathway desirable for adaptive (advantageous) growth of myocardium and prevention of apoptosis triggered by acute hemodynamic stress [33], which leads to the myocardial hypertrophy and heart failure.

MicroRNA-27a passenger strand (miRNA-27a*) is upregulated in non-infarcted areas and in the border zone of the post MI heart as well as in extracellular vesicles (EV) secreted by cardiac fibroblasts and detected in plasma of CHF rats. One of the miRNA-27a* targets is LIM domain 5 (PDLIM5), a gene which has been shown to play a major role in cardiac structure and function (Figure 2) [27,41]. MiRNA-27* is therefore a potential novel therapeutic target in chronic heart as its inhibition could result in favorable PDLIM5 upregulation [27].

MiR-93 has been reported as a negative regulator of the immune response [49]. It also exhibits long-term protective roles via elevating angiogenesis atherosclerosis-associated cardiovascular disease such as myocardial infarction, stroke, and peripheral arterial disease [50]. It has been found that miR-93 is decreased and LIM domain kinase 1 (LIMK1), a serine/threonine kinase, is increased in CHF rats. Upregulation of miR-93 inhibited LIMK1, small GTPase RhoA (RhoA), and Rho kinase 1 (ROCK1) expression in CHF rats. Malondialdehyde (MDA) level was decreased while SOD and Total Antioxidant Capacity Colorimetric (T-AOC) levels were increased by up-regulation of miR-93 or downregulation of LIMK1 (Figure 2). MiR-93 mimics attenuated swelling, vacuolization, mitochondrial lysis and rupture, muscle fiber arrangement disorder, or even rupture of cells in CHF rats under TEM. MiRNA-93 was found to improve ventricular remodeling and reduce cardiac dysfunction, inflammatory markers, and indicators of apoptosis in rats with congestive heart failure [32].

Injection of anti-miR-195-5p in mice model of HF (TAC model of pressure overload) resulted in improved cardiac function and ameliorated inflammation, oxidative stress, and cardiomyocyte injury damage. The possible mechanisms behind that were upregulation of Chemokine receptor type 4 (CXCR4), the direct target of miR-195-5p, enhancement of superoxide dysmutase SOD, inhibited in TAC group, and subsequent inhibition of Janus kinase/signal transducer and activator of transcription (JAK/STAT) signaling pathway activation (Figure 2) [34].

In addition, a study was performed on induced oxidative stress in H9c2 myocardial cells to validate the anti-hypertrophy and anti-apoptosis effects of YQFM Yiqifumai (YQFM) injection, a traditional Chinese drug, derived from Shengmaifang, composed of *Panax ginseng*, *Ophiopogon japonicas*, and *Schisandra chinensis* [51]. In this study, a microarray-based approach was employed to investigate the miRNA expression profiles of YQFM in treating chronic heart failure and found out differentially reversed miRNAs regulated by YQFM: miR-21-3p, miR216-5p, miR219a-2-3p, miR381-3p, miR466c-5p, miR542-3p, and miR-702-5p [31]. It was hypothesized that these microRNAs may be responsible for counteracting anti-hypertrophic and anti-apoptotic effects of oxidative stress in heart muscle.

### 3.5. Apoptosis

Neural precursor cell expressed, developmentally downregulated 4-2 (NEDD4-2)/tropomyosin receptor kinase A (TrkA)/cyclic adenosine 3′,5′-monophosphate (cAMP) axis is described as playing role in degradation and apoptosis of cardiomyocytes. miR-454 activated the cAMP pathway via the NEDD4-2/TrkA/cAMP axis, which was found to be suppressed in HF, and alleviated myocardial damage in rats. miR-454 was aberrantly downregulated in the context of HF, while recent evidence suggests NEDD4-2 in cardiomyocytes. miR-454 exerted anti-apoptotic and protective effects on cardiomyocytes through inhibition of NEDD4-2, while NEDD4-2 stimulated ubiquitination and degradation of TrkA protein. Furthermore, miR-454 activated the cAMP pathway via the NEDD4-2/TrkA axis, which ultimately suppressed cardiomyocyte apoptosis and attenuated myocardial damage. Taken together, the key findings of the study highlight the cardioprotective role of miR-454, which is achieved through activation of the cAMP pathway by impairing NEDD4-2-induced TrkA ubiquitination [35].

### 3.6. Human Studies

To date, only one study regarding microRNA in heart failure was conducted on humans. Ramachandran, S. et al. (2017) [36] found that miR129-5p circulating in microvesicles is a sensitive and specific biomarker for heart failure in pediatric patients. The levels of miR129-5p were inversely related to the degree of clinical severity of heart failure assessed by Ross score in patients with univentricular heart congenital defect. At the same time, the authors describe that miR129-5p levels are downregulated in HL1 cells (cardiac muscle cell line) and human embryonic stem cell-derived cardiomyocytes exposed to oxidative stress. The authors demonstrated that bone morphogenetic protein receptor 2, which has been implicated in the development of pulmonary vascular disease, is a target of miR129-5p, and conversely regulated in response to oxidative stress in cell culture. Further studies are warranted in order to better understand the targets affected by miR129-5p and responsible for the development of heart failure in patients with univentricular physiology [36].

Targeting high mobility group box-1 (HMGB1) via transfection of miRNA-129-5p is yet another mechanism in which miRNA was found to protect heart muscle tissue. Upregulation of HMGB1 and downregulation of miRNA-129-5p was observed in patients with chronic heart failure. The mechanism was confirmed based on transfection of miRNA-129-5p into the rat models with reduced HMGB1 levels, which led to decrease in oxidative stress and inflammation in cardiac cells [52].

Increase in miRNA-208 expression and decrease in miR-1 expression along with significant decreases in the activity of antioxidant enzymes (CAT, SOD, and Gpx) have also been observed in heterogenic group of cardiovascular disease (CVD) patients. The study group consisted of four types of patients (acute coronary syndrome, myocardial infarction (MI), arrhythmia, and heart failure). The lowest concentration of GPX and SOD along with highest expression of miRNA-208 activity was observed in the group of heart failure. miRNA-1 was significantly downregulated in HF patients in comparison to other groups [37].

### 3.7. Therapeutic Potential

Both animal and in vivo studies detect key point mechanisms connected to miRNA and provide promising therapeutic targets for patients with heart failure. Multiple literature reports highlight the importance of miRNA further investigation which can reveal new potential therapeutic modalities [27,28,30,41,42].

The key miRNA with promising therapeutic potential based on the preclinical studies include miRNA-15b, miRNA-195, and miRNA-27a*.

#### 3.7.1. miRNA-15b

As previously described, in murine hearts disturbed with Dicer depletion, miRNA-15b was found to be involved with heart failure. Elevated levels of miRNA-15b silence Pim-1 kinase and ADP-ribosylation factor-like 2 (Arl2), which both affect cellular ATP levels and show multipronged damaging effect it has on mitochondrial function. The study showed that injection of LNA (locked nucleic acid)-modified anti-miRNA-15b significantly decreased miRNA-15b levels in Dicer-deleted adult mice hearts. Moreover, anti-miRNA-15b treatment resulted in significant improvement in cardiac function as recorded by echocardiography and MRI. Furthermore, Hematoxylin/Eosin staining showed histopathological changes such as hypertrophied myofibers and myocyte disarray which were not observed in tissues of animals treated with anti-miRNA-15b. Altogether, these data point towards the hypothesis that strategies targeting the management of elevated miRNA-15b could be one of the potential therapeutic approaches in patients with HF [26].

#### 3.7.2. miRNA-195

Another study provided substantial evidence that miR-195-5p, JAK2, and STAT1 expression were raised and CXCR4 expression was degraded in myocardial tissues of mice with HF (transverse aortic constriction, TAC model of pressure overload). The positive, alleviating impact of anti-miR-195-5p was found both in imaging modalities, biochemistry, and histopathology. The echocardiographic assessment proved decreased left ventricular end-systolic diameter (LVESD), left ventricular end-diastolic diameter (LVEDD), interventricular septal thickness at systole (IVSS) and left ventricular posterior wall thickness in systole (LVPWS) as well as enhanced left ventricle ejection fraction (LVEF). The serum tests showed reduced lactate dehydrogenase (LDH), aspartate aminotransferase (AST), creatine kinase (CK) and creatine kinase myocardial band (CK-MB) levels, declined TNF-α, IL-1β, and Angiotensin II levels, and enhanced IL-10. Moreover, the apoptosis of cardiomyocytes was restrained. These multimodal, promising results in mice need to be further studied, and clinical research might be further extended for the treatment of HF in human patients [34].

#### 3.7.3. miRNA-27a

miRNA-27a* was found to be upregulated in cardiac fibroblasts and packaged into EV resulting in a hypertrophic phenotype in mice with HF in a period of 3 weeks after MI. The researchers observed that systemic administration of miRNA-27a* inhibitor decreased cardiac miRNA-27a* and inhibited the expression of hypertrophic genes, partially improving cardiac function. The cardiac function was mainly assessed by echocardiography revealing that stroke volume was significantly improved, and the cardiac output trended to increase. However, cardiac fibroblasts may produce and secrete many different miRNAs, such as miRNA-21 and miRNA-27a, that contribute to cardiac hypertrophy by targeting the same gene. It will be difficult to determine which miRNA dominates in this pathological process and which inhibitors should be used to obtain the highest effectiveness. Moreover, the authors highlight that it is not clear at the present time what dose and route of administration are most effective. The authors predict that it may be more effective to develop a cardiac homing peptide-based EV delivery system for administration of miRNA-27a* inhibitor as therapy for HF [28].

## 4. Conclusions

Multiple microRNA species are involved in oxidative-stress-related processes within the failing heart. Current evidence provides detailed description of specific pathways and microRNAs involved, which could be used in the development of the diagnostic and therapeutic strategies for HF based on microRNA. To date, both microRNA-mimic and microRNA-inhibition in heart failure show potential in strengthening the antioxidant mechanisms and preventing unfavorable myocardial remodeling.

## Figures and Tables

**Table 1:** A list of research studies evaluating out the role of microRNA that play a role in oxidative-stress mechanisms involved in heart failure according to the sections.

No.	miRNA	Ref.	Type of Study ^1^	Techniqe ^2^	Main Conclusion
*3.1 Mitochondrial integrity and function*					
1	miRNA-690	Wang, X. et al. (2017) [24]	animal	RT qRT-PCR	microRNAs were enriched in mitochondria during heart failure, which establishes a link between microRNA and mitochondrion in heart failure.
	miRNA-696				
	miRNA-532-5p				
	miRNA-345-3p				
2	miRNA-122	Shi, Y. et al. (2021) [25]	animal	qRT-PCR with the miRCute Enhanced Fluorescence Quantitative Assay Kit (Tiangen)	miR-122 causes cardiomyocyte apoptosis by inhibiting Hand2 transcription factor and consequently increasing mitochondrial fission. This mechanism likely contributes to heart failure and modulating this pathway could be therapeutically valuable against heart failure.
3	miRNA-15b	Roy, S. et al. (2013) [26]	animal/in vivo	qRT-PCR	Suppression of inducible miRNA-15b can prevent rapid loss of cardiac function in an animal adult heart and can be a key approach worthy of therapeutic consideration.
*3.2 Antioxidant defence*					
4	miRNA-27a	Tian, C. et al. (2018) [27]	animal/in vivo	qRT-PCR	Increased expression of local microRNAs induced by myocardial infarction may contribute to oxidative stress by the inhibition of nuclear factor erythroid 2-related factor 2(NRF2) translation in chronic heart failure.
	miRNA-28-3p				
	miRNA-34a				
5	miRNA-27a *	Tian, C. et al. (2020) [28]	animal/in vivo	qRT-PCR	Cardiac fibroblast-secretion of miRNA27a *-enriched extracellular vesicles (EV) might act as a paracrine signaling mediator of cardiac hypertrophy and may have a potential to become novel therapeutic target.
6	miRNA-24-3p	Shimizu, T. et al. (2020) [29]	animal	RNA-sequencing, western blot, ELISA	Protein kinase R–like endoplasmic reticulum (ER) kinase (PERK)-mediated suppression of miRNAs by sildenafil improves cardiac dysfunction in heart failure.
*3.3 Iron overload and ferroptosis*					
7	miRNA-224-5p	Zheng, H. et al. (2021) [30]	animal	qRT-PCR, Western blot analysis, luciferase reporter assay	Circulatory RNA (circRNA), microRNA(miRNA) and mRNA work in a regulatory network and reveal potential targets for the treatment of heart failure.
	miRNA-296-3p				
	miRNA-671-5p				
	miRNA-1306-5p				
	miRNA-3082-5p				
*3.4 Cardiac hypertrophy and remodelling*					
8	miRNA-21-3p	Zhao, Y. et al. (2018) [31]	animal	miRNA microarray, bioinforma-tic analysis, qRT-PCR, Western blot	The injection of Yiqifumai (YQFM) has a potential effect which alleviates cardiac hypertrophy and apoptosis in chronic heart failure by miRNA expression regulation.
	miRNA216-5p				
	miRNA-219a-2-3p				
	miRNA-381-3p				
	miRNA-466c-5p				
	miRNA-542-3p				
	miRNA-702-5p				
9	miRNA-93	Su, Q. et al. (2019) [32]	animal/in vivo	RT qRT-PCR, ELISA, Western blot	Upregulated miR-93 and downregulated LIM domain kinase 1 (LIMK1) reduce cardiac dysfunction and improve ventricular remodelling in rats with chronic heart failure.
10	miRNA-142-5p	Sharma, S. et al. (2012) [33]	animal/in vivo	qRT-PCR, Western blot	Downregulation of miR-142 is a critical element of adaptive cardiac muscle hypertrophy in response to hemodynamic stress.
	miRNA-142-3p				
11	miRNA-195-5p	Shen, Y. et al. (2020) [34]	animal	RT qRT-PCR	Depleting miR-195-5p and up-regulating Chemokine receptor type 4 alleviates cardiac function injury in mice with heart failure and may be a potential candidate marker and therapeutic target for heart failure.
For Section 3.4 see also research studies no.: 4 [27] and 5 [28].					
*3.5 Apoptosis*					
12	miRNA-454	Wang, Y. et al. (2021) [35]	animal	RT qRT-PCR, Western blot	The cardioprotective role of miR-454 is achieved through activation of the cyclic adenosine 3′5′-monophosphate(cAMP).
*3.6 Human studies*					
13	miRNA-129-5p	Ramachandran, S. et al. (2017) [36]	human	isolation of mir129-5p from plasma microvesicles, qRT-PCR	miR129-5p was found to be a sensitive and specific biomarker for heart failure in univentricular heart disease in pediatric patients independent of ventricular morphology or stage of palliation.
14	miRNA-208a	Mohammadi et al. (2021) [37]	human	qRT-PCR	The results on different populations of cardiovascular patients (after myocardial infarction, with arrhythmia, heart failure etc.) showed increases in both oxidative stress, inflammation, apoptotic factors, and in the expression of miR-208a.
*3.7 Therapeutic potential*					
15	miRNA-15b	Roy, S. et al. (2013) [26]	animal/in vivo	qRT-PCR	Suppression of inducible miRNA-15b can prevent rapid loss of cardiac function in an animal adult heart and can be a key approach worthy of therapeutic consideration.
For Section 3.5 see also research studies no.: 5 [28] and 11 [34].					

^1^ Types of study include: review, human—study based on human participants, animal—study based on animal models, in vivo—study based on research inside the organism. ^2^ qRT-PCR—quantitative Real-Time Polymerase Chain Reaction, RT qRT-PCR—Reverse Transcriptase quantitative Real-Time Polymerase Chain Reaction, *—passenger strand.
